# Compact Double-P Slotted Inset-Fed Microstrip Patch Antenna on High Dielectric Substrate

**DOI:** 10.1155/2014/909854

**Published:** 2014-08-05

**Authors:** M. R. Ahsan, M. T. Islam, M. Habib Ullah, W. N. L. Mahadi, T. A. Latef

**Affiliations:** ^1^Department of Electrical, Electronic and Systems Engineering, Faculty of Engineering and Built Environment, Universiti Kebangsaan Malaysia (UKM), 43600 Bangi, Selangor, Malaysia; ^2^Department of Electrical Engineering, Faculty of Engineering, University of Malaya (UM), 50603 Kuala Lumpur, Malaysia

## Abstract

This paper presents a compact sized inset-fed rectangular microstrip patch antenna embedded with double-P slots. The proposed antenna has been designed and fabricated on ceramic-PTFE composite material substrate of high dielectric constant value. The measurement results from the fabricated prototype of the antenna show −10 dB reflection coefficient bandwidths of 200 MHz and 300 MHz with center resonant frequency of 1.5 GHz and 4 GHz, respectively. The fabricated antenna has attained gains of 3.52 dBi with 81% radiation efficiency and 5.72 dBi with 87% radiation efficiency for lower band and upper band, respectively. The measured E- and H-plane radiation patterns are also presented for better understanding. Good agreement between the simulation and measurement results and consistent radiation patterns make the proposed antenna suitable for GPS and C-band applications.

## 1. Introduction

In the past couple of years, the emerging trends of wireless and mobile communications technology always requested optimum utilization of the productive resources by ensuring multiple quality services with a single device component. The expected scenario is certainly directed towards the size reduction of the multiple frequency band antennas of low profile, enhanced portability, and multifunctionality [1–4]. Nevertheless, with the increase of frequency bands, the design complexity associated with antenna also intensified. To fulfill the great demand of multifrequency operations in a single component for various wireless communication services, high performance antennas with desired radiation properties have to be developed. For the designing of communication module, it is common to integrate own antenna subsystem which can reciprocate certain standard requirements by wireless system. However, implementing and/or integrating more than one function in the single communication system may help in cost minimization and size reduction of the complete module. For outdoor environment, the position data of an object are given by the global positioning system (GPS) based on the satellite navigation system [5]. Integrating the GPS and C-band satellite frequency in a single antenna module may help in developing centralized remote monitoring system and thus may improve the robustness and efficiency in tracking/monitoring the position of flying aircrafts.

The microstrip patch antennas provide the conveniences through low cost, ease of manufacturability, easy integration, and adaptability with integrated circuit technologies. However, its main weakness is linked with narrow band service [6–8]. This can be overcome by implementing various band widening and size reduction techniques as reported by many researchers. Besides the wide bandwidth and low profile, the antenna has to be cost effective, offer steady radiation patterns, and provide consistent gain for multiband operations. Extensive research works have been carried out in the past years regarding the applications and technologies accompanying the multiband antenna design. By studying ample numbers of research articles, wide variations have been observed as expected in terms of geometrical configurations, size, substrate materials, manufacturing techniques, and analysis methods. A wide range of methods are reported in literatures for achieving reduced antenna size and obtaining more than one frequency band of operations. Some of the techniques involve using rectangular slotted patch [9], circular ring [10], defected ground plane [11, 12], metamaterials [13, 14], electromagnetic band-gap [15], high dielectric substrate [16, 17], magnetodielectric material [18], fractal shape [19, 20], split ring [21, 22], various feeding techniques [23–25], stacked arrangement [26, 27], and optimization technique like genetic algorithms [28, 29]. None the less, still there is room for further development of techniques to enhance the design simplicity, flexibility of operations, and tunable functionality of multifrequency besides maintaining the desired antenna properties for the implementation of the compact wireless device.

This paper proposes a 30 × 35 × 1.905 mm^3^  (*W* × *L* × *h*) rectangular patch antenna loaded with double-P slots and fed by a 7.5 mm long inset microstrip line. On the basis of the well-established mathematical formulation [30], the initial dimension of the microstrip patch antenna has been estimated for desired frequencies. The optimal dimension of the proposed antenna has been achieved through various simulations in finite element method based 3D full-wave electromagnetic high frequency structure simulator (HFSS) [31]. The antenna is designed and fabricated on ceramic-polytetrafluoroethylene (PTFE) composite material substrate with *ε*_*r*_ = 10.2, tan(*δ*) = 0.0023. The usage of high dielectric substrate though reduces the operating bandwidth; however, it assists to achieve the required miniaturization profile of the antenna [32, 33]. The fabricated prototype of the proposed antenna has achieved the resonant frequencies at 1.5 GHz and 4.0 GHz with 200 MHz and 300 MHz bandwidth, respectively. The operating bands of the antenna can successfully cover the *L*_1_ GPS (1572 MHz) operating frequencies [34] and C-band applications [21]. The proposed antenna has obtained a peak gain of 3.52 dBi with 81% efficiency and 5.72 dBi with 87% efficiency for lower band and upper band, respectively. The experimental verification has concluded with good agreement between the measured results from fabricated antenna and the simulation results.

## 2. Antenna Design

**Figure 1:**
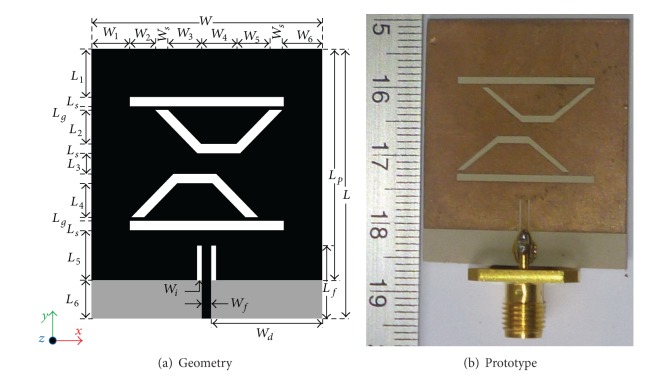
Layout of the proposed antenna (a) geometrical configuration and (b) printed prototype.

Figure 1 represents the fabricated antenna prototype alongside the schematic of the double-P shape slotted inset-fed patch antenna structure. The complete optimized parameters for the proposed antenna are offered in Table 1. Alike typical microstrip antenna, the proposed antenna is made up of a mirrored P-shape slotted radiating patch, a 50 Ω microstrip line inset-fed mechanism, and simple rectangular partial ground plane on the rear side of the substrate. Since the length of the ground plane has a dominant effect on resonant frequency and impedance bandwidth [35], the partial/defected ground plane is chosen by researchers for reduced reflection coefficient and wider gain [36, 37]. The proposed microstrip line inset-fed double-P slotted planar antenna is designed and numerically analyzed by employing HFSS 3D electromagnetic simulator, which is based on the frequency domain solver. The final optimized design of the antenna is printed on a 1.905 mm thick ceramic-PTFE composite material substrate with *ε*_*r*_ = 10.2, tan*(*δ*)* = 0.0023, and dimensions of 30 × 35 mm^2^ (*W *×* L*) by means of in-house printed circuit board (PCB) prototyping machine. The geometrical configuration of the radiating patch element is estimated and optimal parameters are being searched through the use of electromagnetic simulator. To cope up with the expected multifrequency operations, the augmented measurements for the ground plane and dielectric substrate are chosen wisely. A microstrip line of 7.5 mm long and 1.5 mm wide inset-feeding mechanism is selected to connect the radiating patch along with the partial ground plane of 5 × 30 mm^2^ through a 50 Ω coaxial probe at the center of* x*-axis and along the* y*-axis. The microstrip line is copper imprinted alongside with the patch on the substrate as the radiator. The details of the proposed antenna parameters are tabulated in Table 1.

## 3. Experimental Verifications

**Figure 2:**
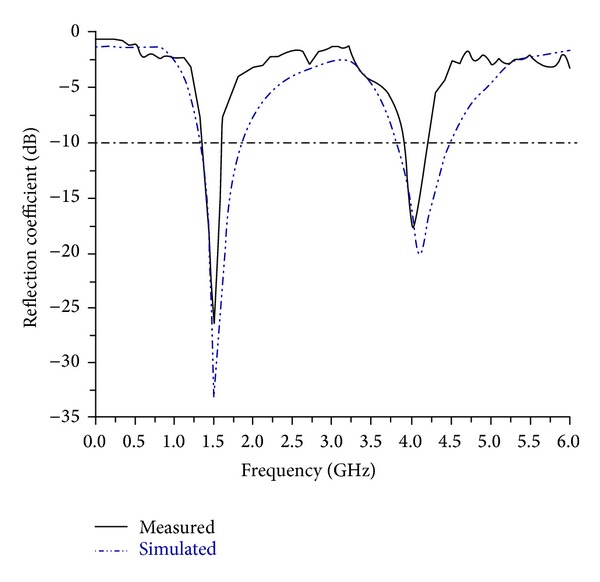
Predicted and experimented reflection coefficient of the proposed antenna prototype.

**Figure 3:**
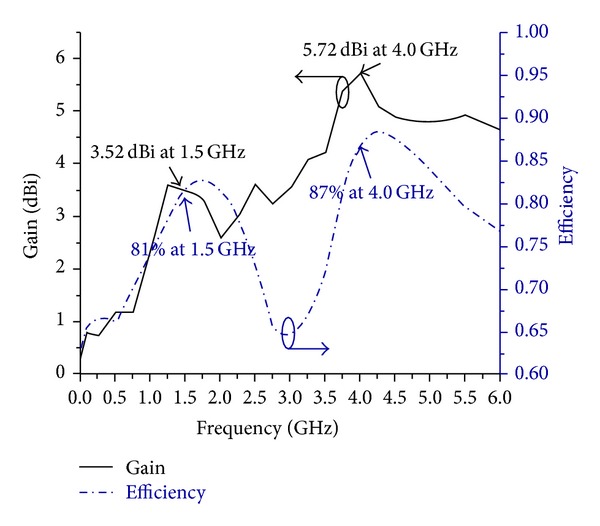
Achieved gain and radiation efficiency of the proposed antenna.

**Figure 4:**
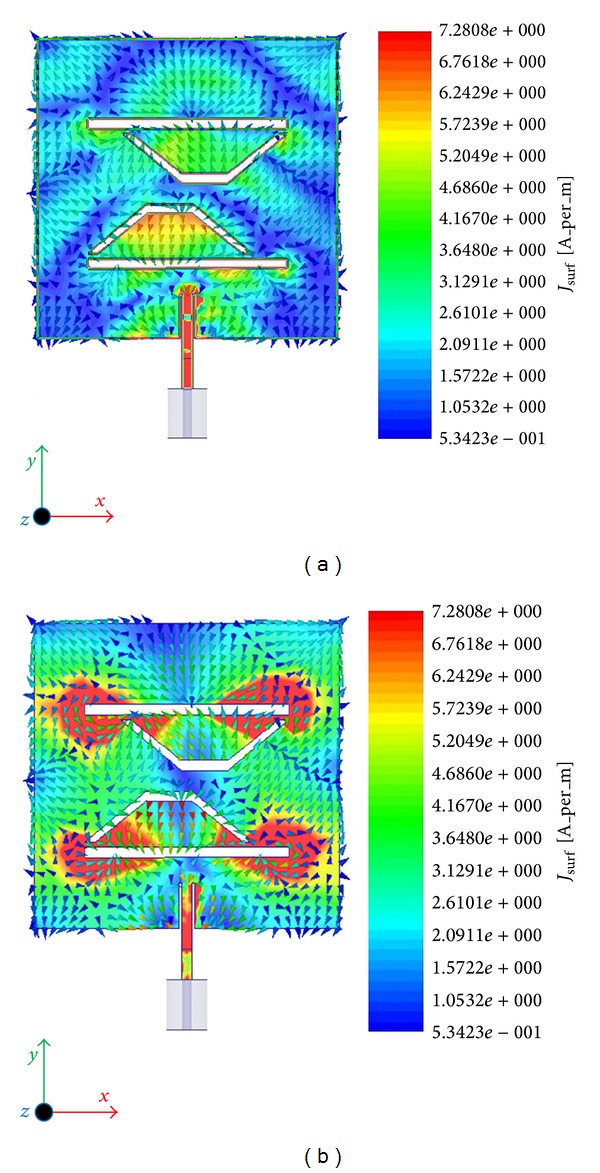
Surface current distribution at (a) lower and (b) upper resonant frequencies of the proposed antenna.

The experimental prototype of the proposed antenna has been fabricated using printed circuit board for verifying and comparing its performance results with the expected one from numerical simulation. Consequently, the PCB model of the antenna is tested in a typical anechoic antenna measurement chamber system with a horn antenna as a reference [38].  Figure 1(b) shows the picture of the PCB model of the proposed antenna for dual band operations. Simulated and measured reflection coefficient versus frequency is shown in Figure 2. The measured reflection coefficient exhibits the operating bands, from 1.35 GHz to 1.55 GHz and from 3.9 GHz to 4.2 GHz at lower and upper bands, consecutively. There is a little dissimilarity between simulated and measured reflection coefficient observed and it can be due to the fringing effect caused by the SMA soldering imperfection. The achieved gain and radiation efficiency of the antenna are demonstrated in Figure 3. It has been realized that average gain is 3.49 dBi with 80% (0.80) radiation efficiency in the lower band and 5.40 dBi with 85% (0.85) in the upper band, correspondingly. At the lower and upper resonant frequencies 3.52 dBi and 5.72 dBi gains and 81% and 87% radiation efficiencies have been achieved, respectively. As the gain of the radiating structure is proportional to the concentration of the surface current, it can be further validated by the electric field distribution illustrated in Figure 4. It can be evidently perceived that the intensity of the flowing current is comparatively lower than upper band. Similarly, as observed in gain profile, the radiation is much stronger in the upper band compared to the lower band.

**Figure 5:**
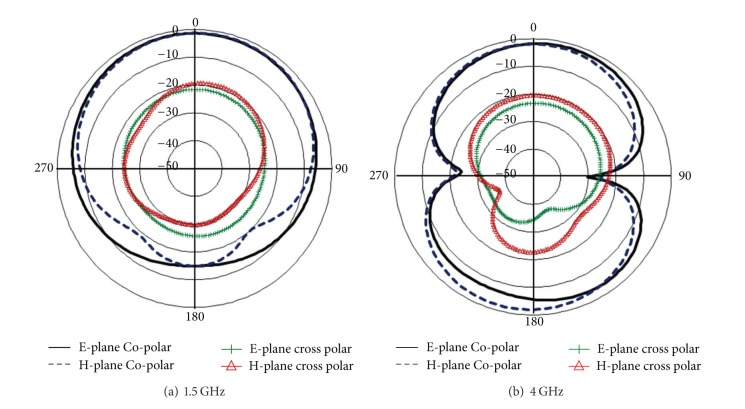
Measured radiation profile of the fabricated antenna.

**Figure 6:**
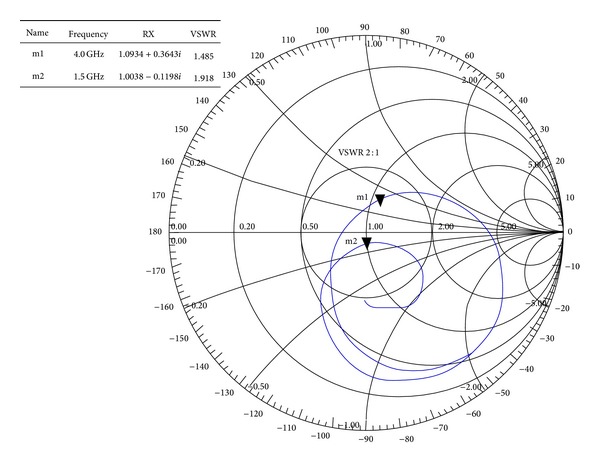
Smith chart of the proposed antenna.

From the surface current distribution outline, the resonant characteristics can also be realized. The lower resonant frequency is obtained through the slots on the lower edge of the radiating patch; particularly “P” shaped slot closed to the microstrip feed line, whereas upper edge of the radiating patch radiates minimum at the lower resonance. Furthermore, the upper edge, especially around the cutting edge of inverse “P” slot, is responsible for the higher resonant frequency. The measured radiation pattern of the proposed antenna is demonstrated in Figure 5. Symmetric and nearly stable radiation profiles at both lower and upper resonance have been realized. The cross polar effects at both resonant frequencies are comparatively lower. A Co-polar −3 dB half power beam width (HPBW) of 109° (54°-0°-304°) in E-plane and 95° (54°-0°-318°) at 1.5 GHz has been measured at broadside direction. Furthermore, at the higher resonance of 4.0 GHz HPBW of 71° (48°-0°-336°) in E-plane and 65° (26°-0°-330°) in H-plane has been observed. However, a little back lobe radiation has been noticed at 4.0 GHz. A considerable amount of back lobe radiation is being observed and possible reason behind this may be the utilization of small sized partial ground plane. Full ground plane may reduce the back lobe; conversely, this may affect the resonant frequencies, gain, and bandwidth of the proposed antenna which is undesirable. Furthermore, this study has been done for achieving certain resonant frequencies; however, still there are possibilities in finding the proper solution for reduced back lobe. Input impedance and VSWR of the proposed antenna can be realized from the smith chart as shown in Figure 6. Both operating bands lie inside the VSWR 2 : 1 circle; the input impedance is close to 50 Ω. The lower resonant frequency lies below the zero line which is capacitive and the upper resonant frequency lies above the zero line, that is, inductive.

## 4. Conclusion

This paper demonstrates the development of inset-fed rectangular microstrip patch antenna with double-P slots. The printed planar antenna has gained the operating frequencies of 1.5 GHz and 4 GHz, which can be utilized for GPS operating frequency and C-band applications. The proposed antenna has achieved bandwidths of 200 MHz and 300 MHz with gain of 3.52 dBi and 5.72 dBi, and radiation efficiency of 81% and 87% for lower band and upper band, respectively. The experimental results for fabricated antenna show good agreement with the simulation results obtained from commercially available finite element based simulator HFSS.

## Figures and Tables

**Table 1:** Optimal dimensions of the proposed antenna.

Parameters	*W*	*W*_1_	*W*_2_	*W*_3_	*W*_4_	*W*_5_	*W*_6_	*W*_*s*_	*W*_*i*_	*W*_*f*_	*W*_*d*_	*L*	*L*_*p*_	*L*_1_	*L*_2_	*L*_3_	*L*_4_	*L*_5_	*L*_6_	*L*_*s*_	*L*_*g*_	*L*_*f*_
Dimension (mm)	30	5	3.5	4.5	4.5	4.5	5	1.5	0.25	1.5	14	35	30	7	4.25	3	4.25	7	5	1	0.25	9.5

## References

[1] Anguera J, Puente C, Borja C (2001). A procedure to design stacked microstrip patch antennas based on a simple network model. *Microwave and Optical Technology Letters*.

[2] Ahsan MR, Islam MT, Ullah MH (2014). A compact multiband inverted a-shaped patch antenna for WiMAX and C-band. *Microwave and Optical Technology Letters*.

[3] Pozar DM (1992). Microstrip antennas. *Proceedings of the IEEE*.

[4] John M, Ammann MJ (2006). Integrated antenna for multiband multi-national wireless combined with GSM1800/ PCS1900/IMT2000 + extension. *Microwave and Optical Technology Letters*.

[5] Chen S, Liu G, Chen X, Lin T, Liu X, Duan Z (2010). Compact dual-band GPS microstrip antenna using multilayer LTCC substrate. *IEEE Antennas and Wireless Propagation Letters*.

[6] Ojaroudi M, Ojaroudi N, Ghadimi N (2013). Enhanced bandwidth small square slot antenna with circular polarization characteristics for WLAN/WiMAX and C-band applications. *Applied Computational Electromagnetics Society Journal*.

[7] Ahsan MR, Islam MT, Ullah MH, Misran N (2014). Bandwidth enhancement of a dual band planar monopole antenna using meandered microstrip feeding. *The Scientific World Journal*.

[8] Anguerat J, Daniel JP, Borja C (2010). Metallized foams for antenna design: application to fractal-shaped sierpinski-carpet monopole. *Progress in Electromagnetics Research*.

[9] Buerkle A, Sarabandi K, Mosallaei H (2005). Compact slot and dielectric resonator antenna with dual-resonance, broadband characteristics. *IEEE Transactions on Antennas and Propagation*.

[10] Gupta SK, Sharma M, Kanaujia BK, Gupta A, Pandey GP (2014). Triple band annular ring loaded stacked circular patch microstrip antenna. *Wireless Personal Communications*.

[11] Hosseini SA, Atlasbaf Z, Forooraghi K (2008). Two new loaded compact planar ultra-wideband antennas using defected ground structures. *Progress in Electromagnetics Research B*.

[12] Andújar A, Anguera J (2012). Multiband coplanar ground plane booster antenna technology. *Electronics Letters*.

[13] Ntaikos DK, Bourgis NK, Yioultsis TV (2011). Metamaterial-based electrically small multiband planar monopole antennas. *IEEE Antennas and Wireless Propagation Letters*.

[14] Li Y, Feng Q (2013). A compact tri-band monopole antenna with metamaterial loaded for WLAN/WiMAX applications. *Journal of Electromagnetic Waves and Applications*.

[15] Xie J-J, Yin Y-Z, Wang J, Pan S-L (2012). A novel tri-band circular slot patch antenna with an EBG structure for WLAN/WiMAX applications. *Journal of Electromagnetic Waves and Applications*.

[16] Madhuri RG, Hadalgi PM, Hunagund PV (2011). Design of high-permittivity rectangular dielectric resonator antenna. *Microwave and Optical Technology Letters*.

[17] Ullah MH, Islam MT (2013). A compact square loop patch antenna on high dielectric ceramic-PTFE composite material. *Applied Physics A: Materials Science & Processing*.

[18] Buerkle A, Sarabandi K A circularly polarized magneto-dielectric resonator antenna with wideband, multi-resonant response.

[19] Saluja N, Khanna R (2012). A novel method to improve current density in multiband triangular fractal antenna. *Electronics and Electrical Engineering*.

[20] Anguera J, Puente C, Borja C, Soler J (2007). Dual-frequency broadband-stacked microstrip antenna using a reactive loading and a fractal-shaped radiating edge. *IEEE Antennas and Wireless Propagation Letters*.

[21] Yusop SH, Misran N, Islam MT, Ismail MY (2012). Design of high performance dual frequency concentric split ring square element for broadband reflectarray antenna. *Applied Computational Electromagnetics Society Journal*.

[22] Li D, Xie YJ, Wang P, Yang R (2007). Applications of split-ring resonances on multi-band frequency selective surfaces. *Journal of Electromagnetic Waves and Applications*.

[23] Islam MT, Shakib MN, Misran N (2009). Design analysis of high gain wideband L-probe fed microstrip patch antenna. *Progress in Electromagnetics Research*.

[24] Wang Z, Fang S, Fu S, Jia S (2011). Single-fed broadband circularly polarized stacked patch antenna with horizontally meandered strip for universal UHF RFID applications. *IEEE Transactions on Microwave Theory and Techniques*.

[25] Zhou Y, Chen C-C, Volakis JL (2007). Dual band proximity-fed stacked patch antenna for tri-band GPS applications. *IEEE Transactions on Antennas and Propagation*.

[26] Anguera J, Puente C, Borja C, Delbene N, Soler J (2003). Dual-frequency broad-band stacked microstrip patch antenna. *IEEE Antennas and Wireless Propagation Letters*.

[27] Ullah MH, Islam MT, Jit MS, Misran N (2012). A three-stacked patch antenna using high-dielectric ceramic material substrate. *Journal of Intelligent Material Systems and Structures*.

[28] Rahmat-Samii Y, Michielssen E (1999). *Electromagnetic Optimization by Genetic Algorithms*.

[29] Jayasinghe J, Anguera J, Uduwawala D (2013). Genetic algorithm optimization of a high-directivity microstrip patch antenna having a rectangular profile. *Radioengineering*.

[30] Balanis CA (2005). *Antenna Theory: Analysis and Design*.

[31] ANSYS *High Frequency Structural Simulator (HFSS)*.

[32] Wong K-L (2004). *Compact and Broadband Microstrip Antennas*.

[33] Peng Z, Wang H, Yao X (2004). Dielectric resonator antennas using high permittivity ceramics. *Ceramics International*.

[34] Ma S-L, Row J-S (2011). Design of single-feed dual-frequency patch antenna for GPS and WLAN applications. *IEEE Transactions on Antennas and Propagation*.

[35] Nguyen MT, Kim B, Choo H, Park I Effects of ground plane size on a square microstrip patch antenna designed on a low-permittivity substrate with an air gap.

[36] Weng LH, Guo Y-C, Shi X-W, Chen X-Q (2008). An overview on defected ground structure. *Progress in Electromagnetics Research B*.

[37] Azim R, Islam MT, Misran N (2011). Ground modified double-sided printed compact UWB antenna. *Electronics Letters*.

[38] Blake LV, Long MW (2009). *Antennas: Fundamentals, Design, Measurement*.

